# Long-range DNA interactions: inter-molecular G-quadruplexes and their potential biological relevance

**DOI:** 10.1039/d2cc04872h

**Published:** 2022-10-19

**Authors:** Denise Liano, Ludovica Monti, Souroprobho Chowdhury, Federica Raguseo, Marco Di Antonio

**Affiliations:** Imperial College London, Chemistry Department, Molecular Sciences Research Hub 82 Wood Lane W12 0BZ London UK m.di-antonio@imperial.ac.uk; The Francis Crick Institute 1 Midland Road NW1 1AT London UK; The Institute of Chemical Biology, Molecular Science Research Hub 82 Wood Lane W12 0BZ London UK

## Abstract

Guanine-rich DNA sequences are known to fold into secondary structures called G-quadruplexes (G4s), which can form from either individual DNA strands (intra-molecular) or multiple DNA strands (inter-molecular, iG4s). Intra-molecular G4s have been the object of extensive biological investigation due to their enrichment in gene-promoters and telomers. On the other hand, iG4s have never been considered in biological contexts, as the interaction between distal sequences of DNA to form an iG4 in cells was always deemed as highly unlikely. In this feature article, we challenge this dogma by presenting our recent discovery of the first human protein (CSB) displaying astonishing picomolar affinity and binding selectivity for iG4s. These findings suggest potential for iG4 structures to form in cells and highlight the need of further studies to unravel the fundamental biological roles of these inter-molecular DNA structures. Furthermore, we discuss how the potential for formation of iG4s in neuronal cells, triggered by repeat expansions in the *C9orf72* gene, can lead to the formation of nucleic-acids based pathological aggregates in neurodegenerative diseases like ALS and FTD. Finally, based on our recent work on short LNA-modified probes, we provide a prespective on how the rational design of G4-selective chemical tools can be leveraged to further elucidate the biological relevance of iG4 structures in the context of ageing-related diseases.

## Abstract 2

Intermolecular G-quadruplex structures can form within distal region of genomic DNA, contributing to chromatin looping. Herein, we discuss recent evidence supporting formation of iG4s in living cells and their potential biological function.

## Graphical abstract

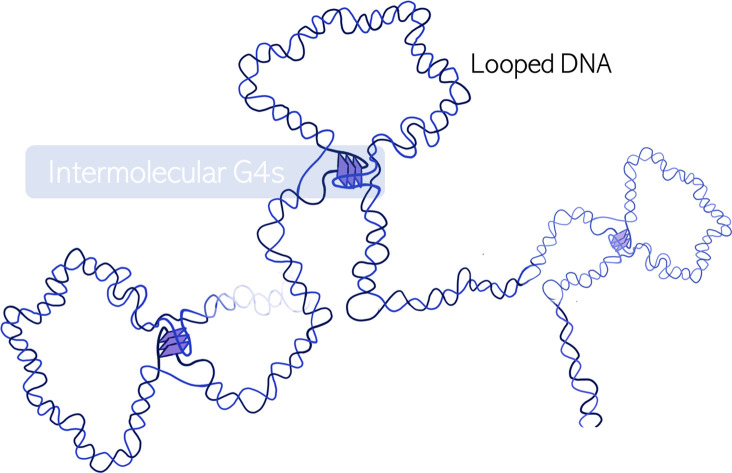

## 1 Introduction

**Fig. 1:**
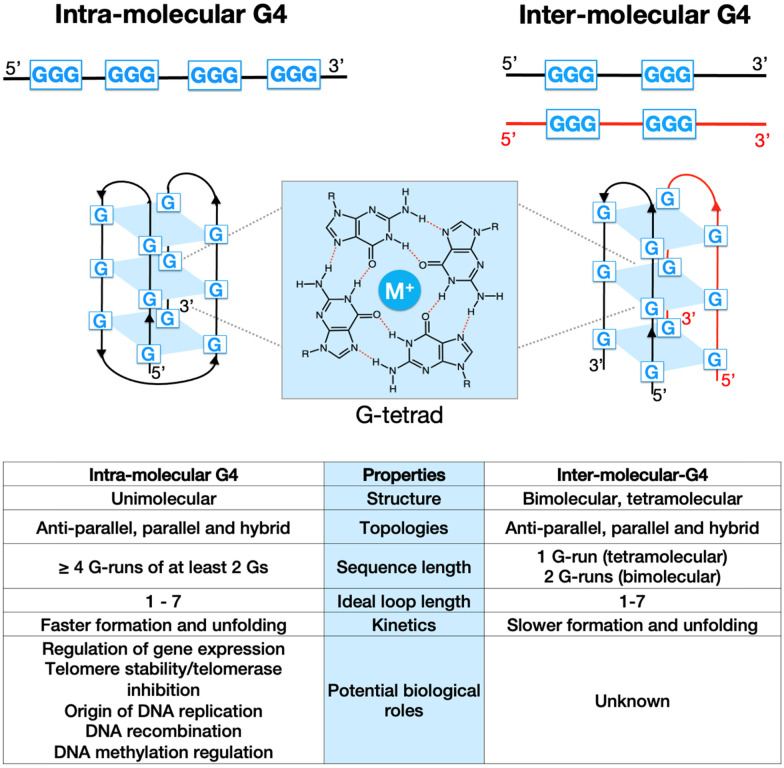
Schematic representation of intra- and inter-molecular G-quadruplex (G4) structures and their properties. A planar G-tetrad structure is formed by four guanine residues stabilised by Hoogsteen hydrogen bonds and coordinated by a central monovalent cation. Intra-molecular G4s are formed within a single DNA strand, while inter-molecular G4s generate from two (or more) strands.

G-quadruplexes (G4s) are non-canonical structures originating from DNA (or RNA) sequences that are rich in guanines (Gs). Under physiological conditions, four Gs can form a planar tetrameric structure know as G-tetrad (Fig. 1), which is stabilised by Hoogsteen hydrogen bonds and coordinated by a monovalent alkali cation, typically K^+^ or Na^+^. Depending on the stoichiometry of formation, G4s can be either intra-molecular (forming from a single strand of DNA), or inter-molecular (forming from multiple DNA strands, iG4s) (Fig. 1). These two G4-structures have shown to have different structural and physio-chemical properties, including kinetics of folding and thermodynamic stability (Fig. 1, table).^1,2^ In addition, the orientation of the nucleic acid strand(s) can produce G4-structures with different topologies, such as parallel, antiparallel, hybrid, and looped.^3^ Bioinformatic and genome-wide sequencing approaches have demonstrated that intra-molecular G4s are highly prevalent in the human genome, with over 700 000 detected structures that are particularly enriched in functional regions, such as gene promoters and telomeres.^4^ It has been therefore proposed that G4s can potentially play a key role in physiological processes in eukaryotes, especially gene-expression regulation.^5^ Furthermore, the presence of G4s in promoters (*e.g.*, *cMYC*) and/or telomers of cancer-related genes has prompted the study of these structures in the context of cancer, leading to the development of G4-ligands as novel potential chemotherapeutic agents.^6,7^

More recent studies on G4s focused on investigating their formation in the genomic context of living cells. In fact, the abundant presence of G4-structures in the human genome, does not necessarily imply their formation *in cellulo*, which is something that required further validation. Immuno-precipitation experiments using a G4-specific antibody allowed the detection of G4s in human chromatin, revealing that these structures act as markers of active transcription and associate with accessible chromatin regions.^8^ These findings encouraged the development of G4-targeting approaches, as well as biological characterization of these unique genomic structures.^9^ Moreover, live cell imaging using small molecule probes have confirmed the formation of G4-structures in living cells under non-perturbative conditions, highlighting the potential of these structures to act as important biological regulators.^10,11^

It is becoming increasingly apparent that G4s can represent a significant structural feature that impacts the overall chromatin architecture and its three-dimensional structure. The ability of chromatin to establish long-range interactions is crucial to regulate gene-expression.^12^ It is therefore conceivable that G4s may play a role in this process through the formation of inter-molecular structures (iG4s) which allows connection between distal DNA sequences in the space. However, when investigating the biological function of these secondary structures, iG4s have been often overlooked and deemed unlikely to be physiologically relevant, due to the low probability of multiple DNA strands to form such multi-molecular architectures in the context of chromatinized DNA. In this context, our laboratory has recently reported the first example of an endogenous human protein that is able to selectively bind to iG4s over equivalent intra-molecular G4-structures, thus supporting the idea that iG4s can be biologically functional in the context of living cells.^13^ In this feature article, we provide an overview of the chemical-biology approaches we have developed in our laboratory to address unanswered questions on G4-biology, with particular interest on iG4s and their potential biological role(s) in the context of ageing-related disease models, such as cancer and accelerating ageing disorders.

## 2 CSB as a selective iG4 protein binder

The possibility of high-ordered G4 topologies to form within multiple single-stranded G-rich sequences initially emerged from the study of telomeric DNA.^14^ In fact, telomeric sequences are constituted of (G_3_T_2_A)_*n*_ repeats in humans, and of (G_4_T_2_)_*n*_ and (G_4_T_4_)_*n*_ repeats in the ciliates *Tetrahymena* and *Oxytricha*, respectively.^15^*Tetrahymena* repeats are very polymorphic and most G-rich repeats fold into topologically heterogeneous conformations in solution, with iG4s being the preferred conformation under physiological conditions.^15–17^ Interestingly, Moye *et al.* reported that the human telomerase is able to recognise and extend telomeric DNA when folded into an iG4 conformation, suggesting that telomeric DNA can potentially assemble into iG4-structures to be recognised as a substrate of telomerase.^18^

Following these observations, our laboratory has recently demonstrated that the human Cockayne Syndrome B (CBS) protein is a selective iG4s interactor.^13^ CSB is a 168 kDa protein which belongs to SWI/SNF family of chromatin remodelling factors. CSB is codified by the *ercc6* gene on chromosome 10q11.^19^ Mutations of the *ercc6* gene are associated with approximately 70% of Cockayne Syndrome (CS) cases.^20,21^ CS patients exhibit a variety of clinical features which cause accelerating ageing and, ultimately, premature death.^22^ The pleiotropic phenotype of this disease derives from the diverse roles played by CSB, including chromatin remodelling and transcription-coupled DNA repair (TCR).^19^ Scheibye-Knudsen *et al.* demonstrated that CSB is able to resolve ribosomal DNA (rDNA) G4-structures in an ATP-independent fashion, albeit this protein has never been classified as a canonical helicase.^23–25^ The hypothesis that CSB is essential for the resolution of rDNA G4-structures derives from the observation that G-rich rDNA sequences can form G4s during rDNA transcription,^26,27^ as increased transcriptional pausing has been found at these sites in the absence of functional CSB.^23^

**Fig. 2:**
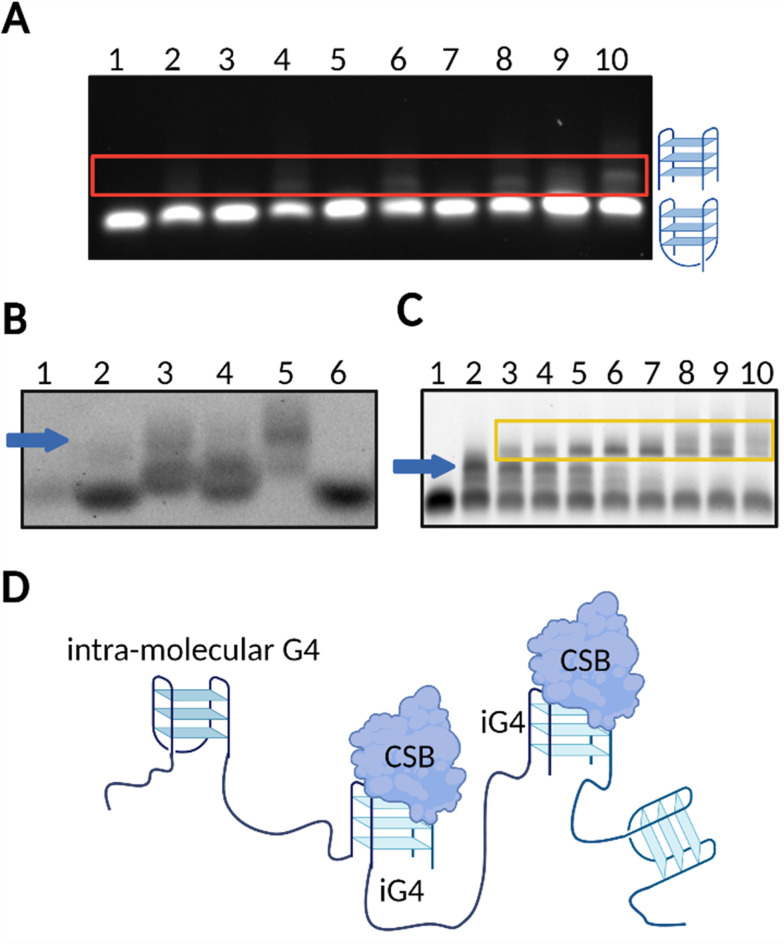
rDNA sequences form iG4s that are selectively bound by CSB. (A) Native gel electrophoresis showing that untailed rDNA is not able to form iG4s in the presence of Li^+^ or K^+^ (lanes 1 and 2, respectively). A tail of at least 3–5 bp is required for iG4s to form under K^+^ conditions (upper bands displayed in lanes 4 and 6, respectively), but not under Li^+^ conditions (lanes 3 and 5). A longer tail of 10–20 bp allows the formation of more stable iG4s which can mostly form in presence of K^+^ (lanes 8 and 10, respectively) compared to Li^+^ conditions (lanes 7 and 9). (B) NMM staining of agarose gel confirmed formation of folded G4 structures from rDNA sequences (lane 2: untailed rDNA in K^+^; lane 3 to 5: tailed rDNAs under K^+^ conditions; lane 6: untailed rDNA in Li^+^). ssDNA remained unstained (lane 1). iG4 bands are indicated with a blue arrow. (C) Agarose EMSA gel on tailed rDNA G4 using increasing concentrations of CSB in K^+^ conditions (lanes 2–10). Lane 1 is a control with the oligonucleotide annealed in Li^+^. iG4 bands are indicated with a blue arrow while the interaction between the iG4 and CSB is indicated within a yellow box. (D) Schematic representation of the selective binding of CSB towards iG4s.^13^

Our group has confirmed that CSB is able to resolve G4 structures formed by a series of rDNA sequences (rDNA-1:5′-GGGGCCGGGGGTGGGGTCGGCGGGGAAA-3′, rDNA-2:5′- GGGTCGGGGGGTGGGGCCCGGGCCGGGG-3′, rDNA-3:5′- AGGGAGGGAGACGGGGGGG-3′), and, interestingly, this effect was also associated to negligible resolution of other types of G4s structures, such as c-KIT1, c-MYC, HRAS, and hTELO.^13^ These findings suggested that, in the presence of a short single-stranded tail (5′-ATAATTATAAATAAATAAT-3′), rDNA G4s might fold into peculiar G4-structures that are specifically recognised and resolved by CSB. Accordingly, we investigated the topology of different rDNA G4s annealed in conditions that either promote (K^+^) or not (Li^+^) G4-formation. For the majority of the rDNA G4s tested, Circular Dichroism (CD) revealed typical spectra of a parallel G4-topology, with a maximum peak at ∼263 nm and minimum peak at ∼240 nm.^13,28,29^ Given that parallel G4s are highly common topologies,^1,9^ we reasoned that it was unlikely that a topology-based selectivity was responsible for the observed selective resolvase activity of CSB.^13^ Indeed, on closer inspection, analysis of the rDNA substrates by native gel electrophoresis revealed that rDNA G4s sequences (rDNA-1, rDNA-2 and rDNA-3) can potentially assemble into iG4-structures in the presence of K^+^ and when a minimum 3-bp overhang (5′-ATA-3′) is added at the 5′- or 3′-end (Fig. 2(A)). To confirm that all the G4-species observed under these conditions could be ascribed to folded G4s, we stained the native gel with the G4-specific probe, *N*-methylmesoporphyrin IX (NMM),^30^ which is a selective fluorescent probe for G4-structures (Fig. 2(B)).^13^ Notably, gel-based binding analysis revealed a selective interaction of CSB with the iG4s formed by the rDNA templates (Fig. 2(C)). The binding affinity of CSB towards the multimeric rDNA conformations was surprisingly high, with a calculated dissociation constant (*K*_d_) in the picomolar range (*K*_d_ = 300–500 pM). This *K*_d_ is comparable to other very high affinity DNA and RNA G4-binding proteins, such as DHX36, which also displays *K*_d_ values in the picomolar range.^31^ In contrast, the binding of CSB towards intra-molecular rDNA G4s required higher concentrations of the protein (>50 nM) to trigger any binding event. Similarly, high concentrations of CSB were required to allow its interaction with established intra-molecular G4s: c-KIT1, c-MYC, HRAS, and hTELO, or unstructured ssDNA, indicating a significantly higher (∼100 fold) binding affinity of CSB towards rDNA iG4s compared to intra-molecular G4s and ssDNA.^13^ Moreover, our data show that CSB is generally able to interact with iG4s and its binding towards these particular G4-structures is not limited to rDNA substrates. Indeed, we have observed remarkable low picomolar affinity (*K*_d_ = 10 pM) against iG4 formed from the *Oxytricha* telomeric sequence d(G_4_T_4_G_4_),^17,32^ confirming the binding selectivity of CSB for iG4s (Fig. 2(D)).

The ability of CSB to bind G4s is not surprising *per se* since, recently, another chromatin remodelling factor, SMARCA4, has been observed to bind intra-molecular G4s with high affinity, suggesting that proteins belonging to SWI/SNF family might contain a conserved domain that can interact with G4 structures.^33^ Moreover, CSB contains a glycine/arginine region at its N-terminal domain, which makes the protein reminiscent of other G4-binding proteins like nucleolin.^34,35^ However, what is surprising is the selective binding towards iG4s that CSB displays and that has never been reported for any other G4-binding protein to date. Although a full structural characterization is still required to fully understand this selectivity, our findings suggest that iG4s might form in cells, opening up to the idea that such structures can also be biologically relevant, something that has always been neglected to date. Importantly, given the very diverse structural architecture of iG4s compared to the more canonical unimolecular G4s, we anticipate that the biological functions of iG4s might be significantly different to what has been reported so far for the “canonical” intra-molecular G4s.

## 3 Formation of cellular iG4s in ribosomal DNA

**Fig. 3:**
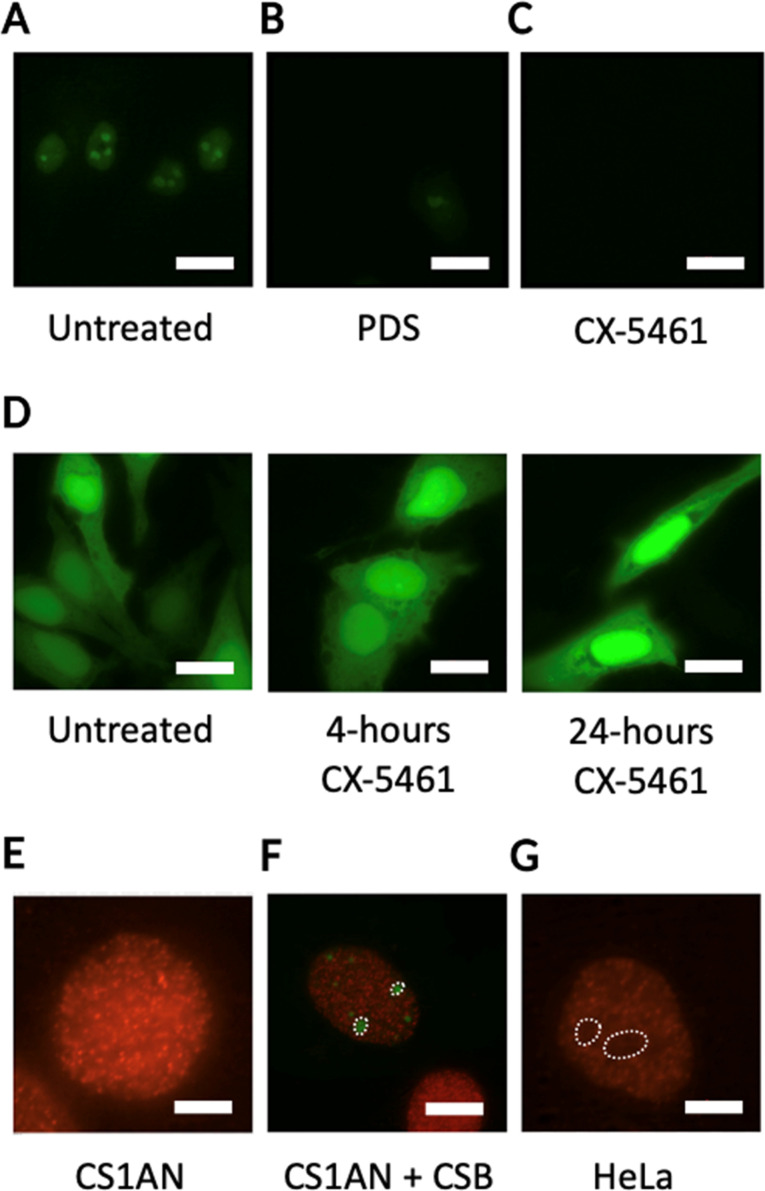
G4-ligands displace CSB from the nucleoli of human cells, while BG4-staining is reduced in the presence of the protein. (A) Localisation pattern of CSB in HeLa cells without treatment (Untreated) or upon 24 hours treatment with either (B) PDS or (C) CX-5461. CSB localisation is followed by fusing the protein with an EGFP tag (green). CSB signal is recorded at 470 nm. White bars indicate 20 μm. (D) Localisation pattern of EGFP in HeLa cells is not influenced by treatment with CX-5461. (E) BG4 immuno-staining in CS1AN cells indicating homogeneous BG4 localisation. BG4 signal is recorded at 590 nm. White bar indicates 5 μm. (F) BG4 immuno-staining in CS1AN cells re-expressing CSB revealed lack of BG4 staining in nucleoli occupied by CSB-EGFP (white circles). CSB signal is recorded at 470 nm, while BG4 signal is recorded at 590 nm. White bar indicates 10 μm. (G) Lack of nucleolar BG4 staining observed in HeLa cells (white circles). BG4 signal is recorded at 590 nm. White bar indicates 5 μm.^13^

The promising results obtained with CSB *in vitro* prompted us to investigate whether the specific interaction of this protein with iG4s could be observed in cells. Cellular localisation studies revealed that CSB is mainly distributed in the nucleoli of eukaryotic cells (Fig. 3(A)),^36^ where primary ribosome biogenesis occurs.^37^ In addition, recent studies demonstrated that CSB is required for rDNA synthesis as a key component of RNA PolI transcription machinery,^38^ with CSB-deficient cells showing increased activity of poly-ADP ribose-polymerase 1 (PARP1).^23,39,40^ Persistent activation of PARP1 leads to premature ageing in *C. elegans* models treated with G4-stabilising ligands, suggesting that the stabilisation of G4s causes stalling of the RNA PolI transcription and activates DNA-damage response pathways.^23^ rDNA genes are arranged in arrays of tandem repeats, resulting in a local concentration of effectors which contributes to generate a dense, G-rich, and transcriptionally active environment that is ideal for iG4-formation.^12^ Therefore, we hypothesised that (i) iG4s could be formed in cells and be bound by CSB, and (ii) treatment with G4-ligands could cause displacement of CSB and induce the observed rDNA transcriptional arrest. To assess whether well-known G4-ligands, such as pyridostatin (PDS)^41^ and CX-5461,^7^ compete with CSB for binding to iG4s and cause CSB-displacement from the DNA structure, we conducted *in vitro* competition-binding assays. After incubation of a pre-bound iG4 rDNA-CSB complex with the G4-ligands, we observed a dose-dependent displacement of CSB from the iG4 substrate. We then asked if a similar displacement of CSB could also be observed in cells. To assess this, we incubated HeLa cells, which endogenously express CSB, with either PDS or CX-5461. Strikingly, treatment of HeLa cells with G4-ligands caused a significant reduction of CSB in the nucleoli (Fig. 3(B) and (C)). Conversely, clear CSB staining was observed in the nucleoli of cells that were not treated with G4-ligands (Fig. 3(A)). These results suggest that G4-ligands can compete with the protein for binding to G4-structures formed within nucleolar rDNA sequences, confirming the potential for iG4s to be formed in cells at the rDNA level.^13^ Interestingly, Lyama *et al.* described the nucleolar displacement of CSB upon treatment of HeLa cells with CX-5461, however, this effect was attributed to a transcriptional inhibitory effect caused by the ligand.^36,42^ In addition, it has been demonstrated that CX-5461 acts as a potent G4-binder and -stabiliser,^7^ suggesting that the displacement of CSB from the nucleoli upon treatment with the ligand is more likely to be caused by binding of the molecule to iG4s rather than by transcriptional regulatory effects, as we also observed with PDS. To validate this, we used immuno-fluorescence to measure changes in expression levels (*i.e.*, transcriptional inhibition) of a green fluorescent protein (EGFP) upon 4 and 24 hours treatment with CX-5461. Notably, we failed to detect any transcriptional inhibition (Fig. 3(D)), which reinforced the hypothesis of CSB being displaced by rDNA iG4s formed in cells, as we also observed *in vitro* and upon treatment of HeLa cells with validated G4-ligands like PDS.

To confirm that CSB is bound to iG4s in the nucleoli of living cells, we performed immunostaining experiments using a synthetic G4-specific antibody, BG4,^8,43^ in Cockayne Syndrome cells (CS1AN) that are genetically impaired of the CSB gene. Immuno-staining of G4s with the BG4 antibody revealed a strong staining in the nucleoli of CS1AN cells, suggesting G4-formation at the rDNA level (Fig. 3(E)). Interestingly, we observed that the re-expression of CSB in CS1AN cells induced CSB-nucleolar localisation and caused a strong reduction of the BG4-staining in the nucleoli of these cells (Fig. 3(F)), suggesting that CSB is bound to iG4s in the nucleoli of living cells.^13^ In addition, we observed a similar lack of nucleolar BG4 staining in HeLa cells that express endogenous CSB. This effect was comparable to what was observed in CS1AN cells that re-express CSB (Fig. 3(F)), confirming the ability of the CSB protein to compete with BG4 antibody for binding to nucleolar iG4s.^13^

**Fig. 4:**
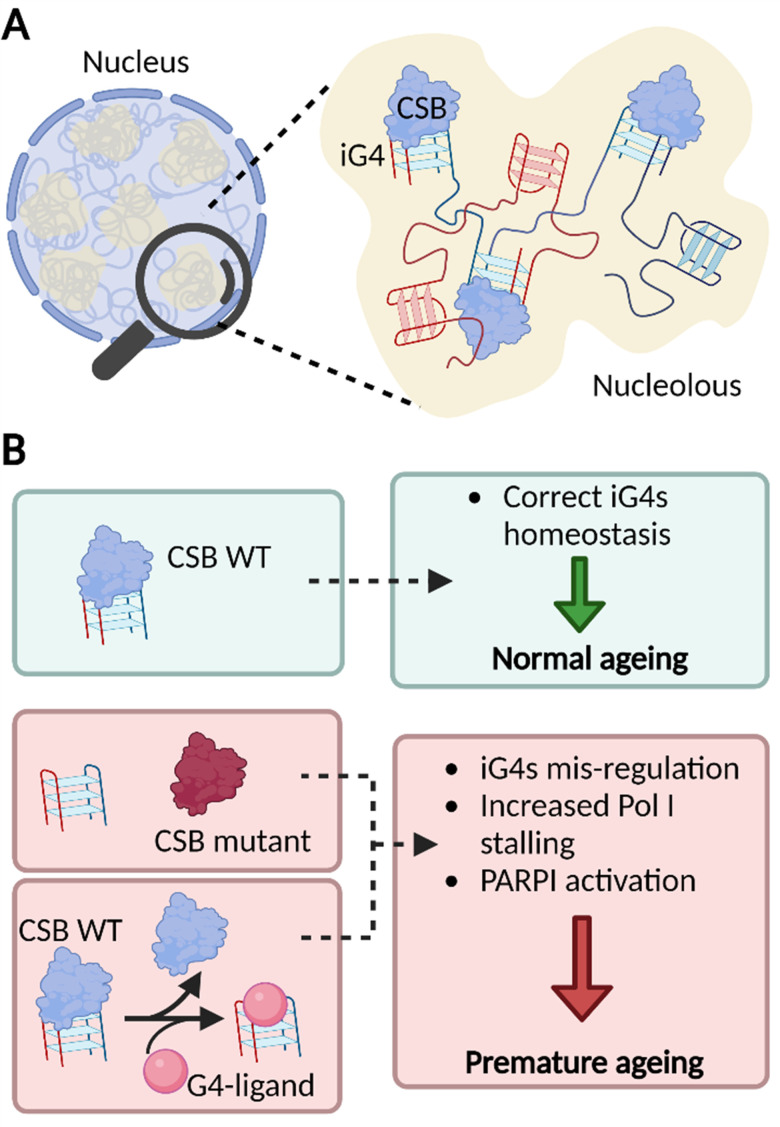
iG4s can form within nucleoli of cells and their homeostasis is regulated by CSB. (A) Schematic representation of a cellular nucleus containing dense regions, referred as nucleoli (in yellow). The nucleolar environment is enriched in rDNA sequences which are prone to interact and form iG4s that are recognised and handled by CSB. (B) Our model suggests that functional CSB binds iG4 structures promoting healthy ageing (green boxes). When CSB is not functional or after treatment with G4-ligands, iG4s are mis-regulated, promote stalling of PolI, and activate PARP1 response. All these effects are known to trigger premature ageing (red boxes).^13,23^

Overall, our findings strongly suggest that iG4s could be formed in the nucleoli of human cells where CSB acts as an endogenous binder of these structures. The dense nature of the nucleoli supports a model where multiple rDNA genes form long-range iG4 interactions (Fig. 4(A)), which might be key to sustain nucleolar cellular homeostasis and of particular importance to the premature ageing phenotypes observed in CS patients that lack functional CSB (Fig. 4(B)).^13^

## 4 The potential role of iG4s in neurodegenerative aggregates

### 4.1 ALS and FTD genetic traits

Amyotrophic lateral sclerosis (ALS) and frontotemporal dementia (FTD) are fatal neurodegenerative diseases that affect the motor system and cognitive behaviors with severe consequences on execution of functions, behaviors, and language abilities. ALS affects the motor neurons in the frontal cortex, brainstem, and spinal cord, while FTD is related to motor neuron disorder. The link between the two diseases can be appreciate both at the clinical level and by the overlapping genetic profiles. In fact, both diseases present mutations in a series of genes and a characteristic expansion of the Chromosome 9 Open Reading Frame 72 gene (C9Orf72).^44^ More specifically, C9Orf72 presents a hexanucleotide repeat expansion (GGGGCC)_*n*_ that is the most common cause of ALS, accounting for approximately 40% of the familiar cases (*i.e.*, family history of ALS) and 7% of sporadic cases in Europe.^45^ Statistical studies have shown that healthy people usually present a repeat number (*n*) of 2, while in ALS patients the *n* value typically raises from hundreds to thousands of repeats.^46^ In addition, a smaller part of the population presents an intermediate number of repeats, from 20 to 30. It has been reported that an expansion of 24 or more (*p* value = 2 × 10^−4^) should be considered pathogenic.^45^

Due to the significant overlap in genetic hallmarks, ALS and FTD often form a neurodegenerative continuum, where both diseases have been observed within same families, or even the same individual.^47^

### 4.2 Pathological aggregates in ALS and FTD

Much like most neurodegenerative diseases, ALS and FTD are characterised by the presence of pathological aggregates.^48^ The mechanism of formation of such aggregates is still widely uncharacterised, but their composition comprises primarily proteins with low complexity domains and nucleic acids (principally RNA).^49^

**Fig. 5:**
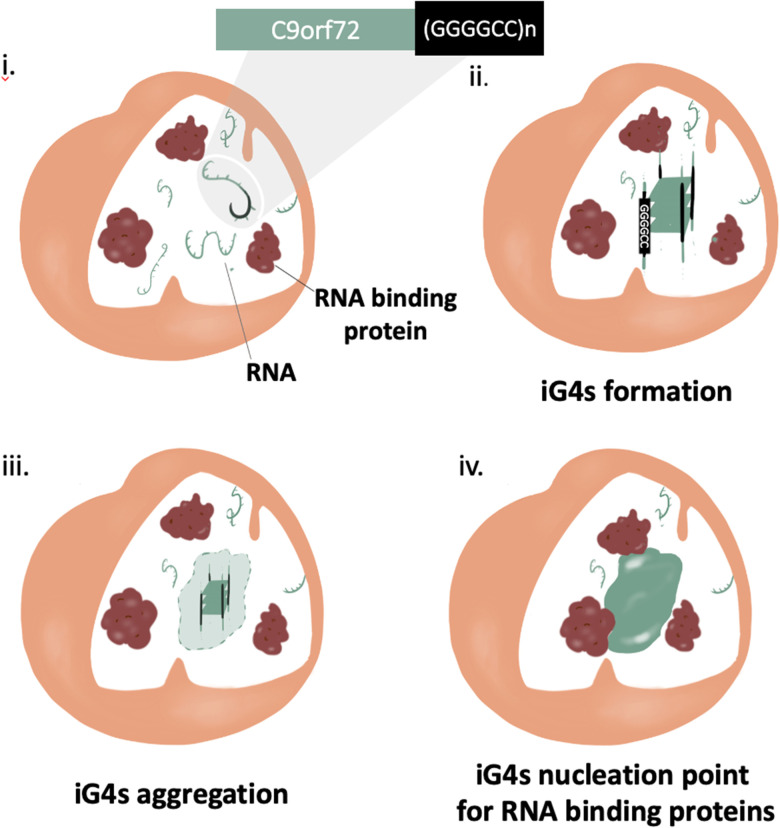
Schematic illustration of iG4s as nucleation triggers for aggregation in ALS/FTD affected cells. (i) ALS and FTD are characterised by a mutation at the C9orf72 gene at the repeat expansion (GGGGCC)n, a sequence with the potential to form iG4s. (i) and (ii) iG4s can aggregate at high concentrations, giving rise to a nucleation point. (iv) RNA binding proteins use the nucleation point created by the iG4s to form pathological aggregates.

Protein aggregation has been considered the main potential trigger for G4^50–52^ and/or condensate formation;^48,53,54^ more recently, increasing evidence highlights the relevance of RNA in such aggregates, due to its ability to stabilise pre-existing protein aggregates through non-specific stacking and electrostatic interactions.^55^ In addition, it is also thought that RNA may play a role in the mechanism of aggregate formation,^56,57^ but this hypothesis is yet to be proved. In this context, Fay *et al.* have shown cell-aggregation of mRNA transcript of C9orf72 gene in ALS affected neurons, proving that nucleic acids can form aggregates.^57^ These results make the C9Orf72 gene an incredibly appealing target to underpin the mechanism of action of nucleic acid in the context of pathological aggregates. Interestingly, the C9Orf72 gene has shown *in vitro* polymorphism to form RNA hairpin and G4s secondary structures, implying that nucleic acid secondary structures might contribute to trigger aggregation. Formation of iG4s within such G-rich nucleic acid repeats would cluster together multiple nucleic strands, providing the potential to the system to aggregate on a microscopic level (nucleation point, Fig. 5). Further structural characterization of the gene (GGGGCC)_*n*_ was performed by Fay *et al.*, confirming the ability of C9Orf72 to form G4s from both RNA and DNA, albeit further elucidation on if and how such G4s might contribute to the aggregation is still required. Moreover, a recent crystallography study has shown the ability of (GGGGCC)_*n*_ to form iG4 DNA:RNA tetrameric hybrids, supporting the hypothesis that this sequence has the potential to form intermolecular links.^58^

Currently, there is no FDA-approved cure for ALS and FTD, although the drugs Riluzole (developed for Parkinson's disease) and Edaravone have shown to increase survival rates in randomized ALS clinical trials.^59,60^ So far, candidate drugs have been developed to target protein–lead aggregation; since recent studies highlight the potential of nucleic acids to potentially be key components of pathological aggregates, there is scope to pursue novel therapeutic pathways that target nucleic acids structures, including iG4s. In fact, it has been shown that G4-targeting ligands have potential to affect the disease progression, although it is not clear how they affect the aggregation pathway.^61^ Considering our recent work that identified an iG4s selective resolvase,^13^ we anticipate that increasing attention should be pointed at these G4-structures, which might be also relevant in the context of neurodegenerative diseases like ALS and FTD.

## 5 Use of LNA probes to disrupt G4s

Our work provides robust evidence to support the idea that G4s can have a range of biological functions that might not simply depend on their genomic location, but also on the stoichiometry of their formation (*i.e.*, iG4s).^12^ G4s might indeed play a significant role in nucleolar homeostasis and could contribute to the formation of pathological aggregates. This highlights the need of chemical probes to selectively target specific G4s within the genome to further elucidate their cellular functions. Furthermore, selective G4-probes that bind to resolve/disrupt G4s (rather than to stabilise them) could represent an alternative therapeutic strategy to alleviate ageing-related diseases like ALS and FTD.^62^ Surprisingly, there is only a limited number of examples of G4-disrupting agents in the literature, highlighting the chemical challenges associated with developing such probes. Waller *et al.* reported one of the earliest examples of the disruption of a DNA G4s, which led to enhanced expression of the *c-KIT* gene in cells upon treatment with such small-molecule.^63^ More recently, Monchaud and co-workers developed an innovative platform to facilitate the identification of small-molecules that can disrupt G4s, and they have shown that G4-disrupting ligands can cooperate with DNA helicases to accelerate G4-unfolding.^64^

A significant drawback of small-molecule G4-ligands is their lack of sequence specificity. The inherent inability of G4-ligands to selectively target specific G4-sequences, together with the high abundance of G4-structures (>700 000 putative G4 sequences) in the human genome, leads to unwanted genome-wide perturbations. This is particularly relevant when considering the diverse biological functions that G4s located in different genomic locations might elicit. For example, a potential G4-targeting based treatment for ALS would require a molecule that selectively recognises G4s formed in the C9Orf72 gene. To this end, a possible alternative to G4-disrupting small-molecules is the use of chemically-modified oligonucleotide probes,^65^ which are ideal sequence-specific G4-disrupting agents as they contain the necessary sequence information *via* Watson-Crick base pairing. Accordingly, early approaches showed the use of Peptide Nucleic Acids (PNA) to disrupt G4s *in vitro*.^66^ Another highly promising chemically-modified alternative are Locked Nucleic Acids (LNA), which have shown to thermodynamically drive greater duplex formation.^67^ LNA-modified probes are particularly attractive for chemical-biology applications due to their enhanced base-pairing, reduced endonuclease degradation, and significantly improved mismatch discrimination, as compared to unmodified DNA.^20,68^

**Fig. 6:**
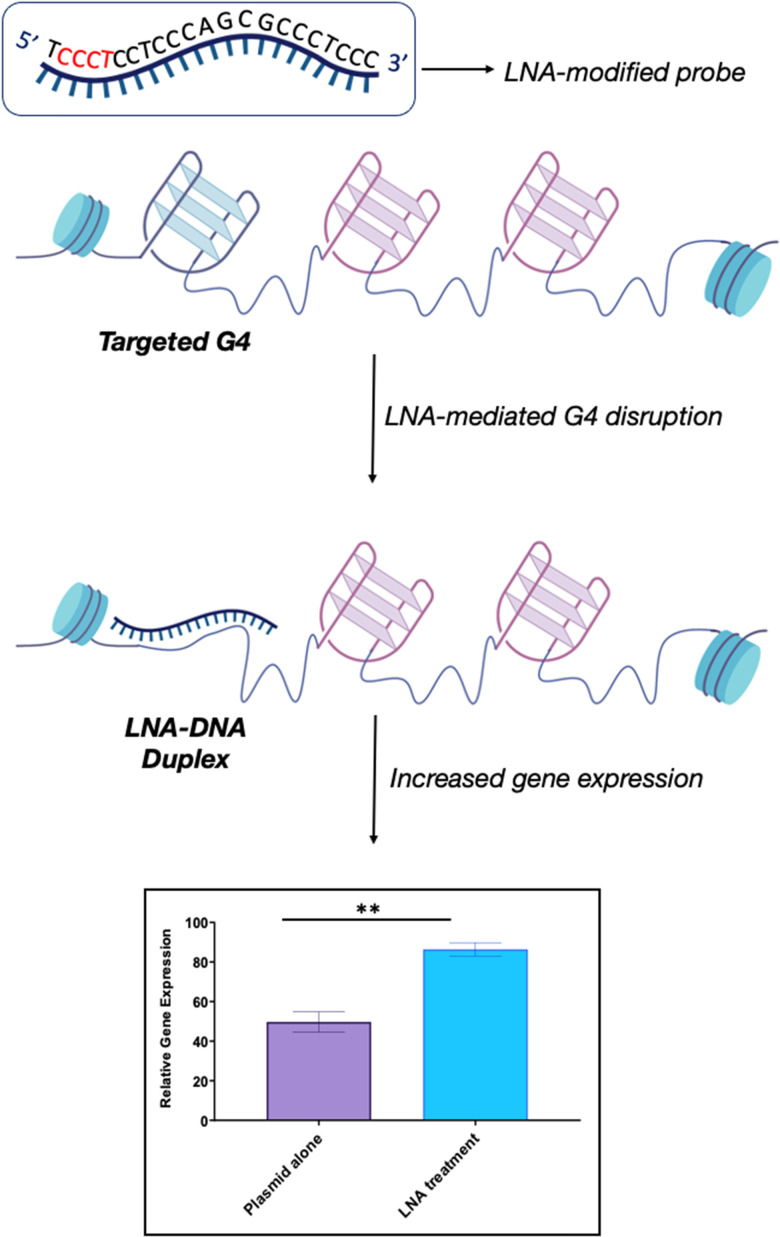
Schematic representation of LNA-mediated G4-disruption. LNA-modified probes contain the necessary sequence information *via* Watson-Crick base pairing to disrupt a specific G4. The position of the LNA-modification along the complementary sequence (in red) can be varied to optimise the G4-disrupting potential of the probe, depending on the requirement. LNA probes can be used to achieve efficient G4-disruption in both biophysical *in vitro* assays and in cells. Our studies revealed that the LNA–DNA complex induces an increase in gene expression without hampering biological processes such as transcription and replication, thus suggesting that this is a viable strategy to achieve G4-disruption in cells.^69^

We recently explored the use of LNA-modified oligonucleotide probes to achieve the disruption of individual G4s. Using a similar approach to the antisense oligonucleotide (ASO) design, we developed a series of LNA-modified cDNA sequences that target model G4 sequences (Fig. 6).^69^ Intriguingly, we found that the G4-disrupting potential of the LNA-modified probes varied significantly depending on the *placement* of the LNA-modification in the complementary sequence. Specifically, probes with the LNAs placed complementary to the Gs (guanines) showed significantly lower G4-disrupting half-lives compared to probes where the LNA modification was in loops or randomly distributed. These observations were consistent across two G4 model systems with different topologies that were tested: a parallel G4 (c-KIT1) and a mixed-type G4 (hTelo). These results suggest that our approach might be an efficient and versatile strategy to achieve disruption of individual G4s with high specificity. Notably, we found that the G4-disrupting potency of the LNA-modified probes was preserved (or even improved) when the length of the sequence was shortened from ∼20 nucleotides to ∼10 nucleotides. These results allowed us to propose the potential application of such short LNA-modified probes beyond their use in *in vitro* biophysical assays. For example, using a combination of single-molecule experiments and biochemical assays, we demonstrated that short LNA-modified probes can disrupt G4s responsible for stalling of DNA polymerases, thus suggesting that these probes could be used to modulate essential biological functions and rescue the progression of the stalled polymerases. Finally, we investigated whether the short G4-targeting LNA probes could be used to modulate gene-expression in cells through a targeted disruption of G4s in promoter regions. Accordingly, we used a previously validated dual-reporter plasmid system^70^ consisting of *renilla* and *firefly* genes. These genes, when expressed, can generate luminescence signals that can be used to quantitatively measure levels of gene-expression. Within the plasmid construct, the promoter of the *renilla* gene is modified to contain the c-KIT promoter sequence, allowing the c-KIT1 G4 present in this promoter to control the expression of the *renilla* gene. On the other hand, the *firefly* gene serves as a control of transfection efficiency between individual experiments. Consistently with our biophysical and biochemical data, we found that short LNA-modified probes significantly enhanced c-KIT1 gene-expression, whereas this effect was not observed when the iso-sequential unmodified DNA probe was used (Fig. 6).^69^

Taken together, our studies demonstrated that short LNA-modified oligonucleotides are viable chemical-biology tools that can individually target and disrupt specific G4s both *in vitro* and in cells. This opens-up new possibilities of the use of these sequence-specific probes to study the biological effect of disrupting individual G4s (rather than using unspecific small-molecules). For example, these probes could be used to study biological phenotypes associated with diseases, such as G4-helicase deficiencies, characteristic of the Cockayne Syndrome, or neurodegenerative diseases such as ALS/FTD that have been linked to the presence of excessive G4-forming repeat-sequences.

## 6 Conclusions

Intra-molecular G4s have been the focus of G4-based research, with remarkable findings showing the importance of these atypical DNA secondary structures in regulating many essential physiological processes, including epigenetic regulation and transcriptional control. In the last decades, highly innovative chemical-biology techniques have been developed to map and visualise the formation of G4s in living cells. However, little attention has been dedicated to study and unravel the biological role of G4-structures formed by multiple DNA-strands (iG4s), which have been deemed not to be biologically relevant due to the low probability of long-range DNA-structures to form in chromatinized DNA.

Our laboratory has recently challenged this dogma and generated new evidence supporting the potential for formation of iG4s in human cells, as well as their possible implication in pathologies like the Cockayne Syndrome. Specifically, our recent work on the CSB protein contributes to demonstrating that iG4s could be physiological components of cellular nucleoli and, in the presence of functional CSB, these structures contribute to cellular homeostasis. In the absence of functional CSB, iG4s accumulate in the nucleoli unresolved, contributing to premature ageing-phenotypes.

The discovery of CSB being an iG4-selective binding protein motivated us to reconsider the role that G4-structures may play in certain diseases, accounting for the potential of also multi-molecular G4-structures (iG4s) to be formed in living cells and contributing to certain phenotypes. For example, in ALS and FTD cells, the accumulation of unresolved iG4-structures might contribute to the formation of the pathological aggregates that are typical hallmarks of these neurological disorders, acting as nucleation points for condensation. These observations lead to re-think the use of molecules to target G4s for therapeutic intervention, and to approach G4-targeting therapies from a fresh perspective. In this context, the use of G4-specific targeting agents, such as LNA-modified oligonucleotide probes, represent a viable alternative to selectively disrupt unresolved, accumulated iG4s and possibly maintain cellular homeostasis. Overall, this feature article aims to provide insights into new strategies and approaches to further investigate iG4s, aiming to better characterise these unique nucleic acid structures and their functional role in human biology, as well as their potential as therapeutic targets in ageing-derived diseases, including neurodegenerative diseases. We anticipate that iG4s will emerge as an important structural feature that contributes towards maintaining chromatin and cellular homeostasis and, therefore, they should be taken into account when investigating G4-biology.

## Author contributions

Conceptualization: M. D. A.; writing – original draft preparation M. D. A., L. M., D. L., F. R. and S. C.; writing – review and editing L. M., and M. D. A. All authors have read and agreed to the published version of the manuscript.

## Conflicts of interest

The authors declare no conflict of interest.

## Supplementary Material
